# Model-based deep learning framework for accelerated optical projection tomography

**DOI:** 10.1038/s41598-023-47650-3

**Published:** 2023-12-08

**Authors:** Marcos Obando, Andrea Bassi, Nicolas Ducros, Germán Mato, Teresa M. Correia

**Affiliations:** 1 Medical Physics Department, Centro Atómico Bariloche and Instituto Balseiro, 8400 Bariloche, Argentina; 2 Dipartimento di Fisica, Politecnico di Milano, Piazza Leonardo da Vinci 32, I-20133 Milano, Italy; 3 University of Lyon INSA-Lyon, Université Claude Bernard Lyon 1, UJM Saint-Etienne, CREATIS CNRS UMR 5220, Inserm, U1294 Lyon, France; 4 IUF, Institut Universitaire de France, Paris, France; 5 Centre of Marine Sciences (CCMAR), Universidade do Algarve, Campus de Gambelas, 8005-139 Faro, Portugal; 6 School of Biomedical Engineering and Imaging Sciences, King’s College London, SE1 7EH London, United Kingdom

**Keywords:** Imaging and sensing, Optical imaging, 3-D reconstruction

## Abstract

In this work, we propose a model-based deep learning reconstruction algorithm for optical projection tomography (ToMoDL), to greatly reduce acquisition and reconstruction times. The proposed method iterates over a data consistency step and an image domain artefact removal step achieved by a convolutional neural network. A preprocessing stage is also included to avoid potential misalignments between the sample center of rotation and the detector. The algorithm is trained using a database of wild-type zebrafish (*Danio rerio*) at different stages of development to minimise the mean square error for a fixed number of iterations. Using a cross-validation scheme, we compare the results to other reconstruction methods, such as filtered backprojection, compressed sensing and a direct deep learning method where the pseudo-inverse solution is corrected by a U-Net. The proposed method performs equally well or better than the alternatives. For a highly reduced number of projections, only the U-Net method provides images comparable to those obtained with ToMoDL. However, ToMoDL has a much better performance if the amount of data available for training is limited, given that the number of network trainable parameters is smaller.

## Introduction

Optical projection tomography (OPT) is often described as the optical analogue of X-ray computed tomography^1^. OPT enables the generation of detailed three-dimensional (3D) images with high (μm) resolution of the optical attenuation (anatomy) and/or fluorescence intensity distribution within transparent (or translucent) samples. OPT works in transmission or fluorescence mode and involves capturing wide-field images of the sample from different angles using a scientific camera, and thereby creating a series of projection images. For transmission data, the sample is illuminated along the optical axis, while for fluorescence OPT, reasonably uniform illumination across the sample is required, regardless of the illumination direction (fluorescence is often detected at a 90° angle relative to the excitation light). The sequential acquisition of each row of pixels of the camera forms a series of sinograms, which consist of 1D images as a function of the rotation angle. Conventionally, the filtered back projection (FBP) method is used to reconstruct 3D images from hundreds of 2D projection images acquired at different angles around the sample.

OPT is used for imaging samples ranging from mm to cm in size, either optically cleared or naturally transparent, including live organisms. However, for *in vivo* imaging, particularly for longitudinal studies, it is essential to reduce the number of acquired projections, and thus reduce the light exposure, to minimise the risk of phototoxicity, a concern for live organisms, and photobleaching of fluorescent proteins^2,3^. This reduction in the number of projections enables *accelerated* (faster) acquisitions, minimising the period of time the organisms are under anaesthesia, as well as the likelihood of movement during the experiment. Unfortunately, according to the classic Shannon-Nyquist sampling theorem, the number of projections required to accurately reconstruct images using FBP should be directly proportional to the number of elements present in the projection images. Therefore, reconstructions from samplings below Shannon-Nyquist conditions (undersampled data) lead to severe streaking artefacts. Nevertheless, different image reconstruction algorithms have been proposed to tackle image degradation that results from sampling below the Shannon-Nyquist conditions. Given that an image can be compressed in some transform domain, its recovery from a smaller number of measurements is guaranteed under compressed sensing theory^4^. Compressed sensing has been successfully applied to OPT^2^ and Magnetic Resonance Imaging (MRI)^5^ to reconstruct high-quality images from undersampled data and thus, accelerate scans. Nevertheless, performance is hindered by the assumptions of compressibility and the slow iterative optimisation reconstruction algorithms.

Over the last decade, deep learning (DL) has demonstrated a paramount performance in image reconstruction problems, providing a useful technique for accelerating image reconstruction from undersampled data in a wide variety of systems^6^. In particular for OPT, Davis et al.^7^ have recently proposed a modified U-Net architecture to estimate streak-free 3D images from FBP reconstructions obtained from undersampled data.

Here, we propose ToMoDL, an unrolled model-based architecture that aims at enhancing both OPT reconstruction and performance time, while providing a framework whose convergence theory has been recently developed^8^. Our proposed method is based on MoDL, a model-based system for arbitrary linear inverse problems that was introduced by Aggarwal et al.^9^. On its basis, an optimisation problem accounting for the forward model in a data consistency term and a convolutional neural network (CNN) as a regularisation prior is solved. Hence, this framework comprises the earliest efforts to combine imaging physics with learned sparsifying transforms^10^. ToMoDL is based on an unrolled architecture, which can be understood in the context of iterative algorithms. For example, in compressed sensing, algorithms alternate between optimisation of a data consistency term and a regularisation term, over multiple iterations, until it converges to an optimal solution. Similarly, an unrolled network combines (model-based) iterative reconstruction with deep learning, and hence, alternates between a data consistency layer and a deep learning-based regularisation block, to learn the iterative reconstruction process directly from the data. More specifically, ToMoDL uses a CNN-based regulariser with shared weights across iterations, and since, in addition, the forward model is explicitly accounted for, the number of network parameters to be learned is significantly reduced compared to direct inversion approaches, thereby providing better performance in training data constrained settings.

As OPT imaging methodologies often rely on custom-engineered setups, the proposed framework introduces different preprocessing alternatives to overcome artefacts produced by potential misalignments between the sample center of rotation and the detector. Using transmission projection images of juvenile zefrafish embryos, we demonstrate the ability of this framework to generate high-quality streak-free 3D images from undersampled data, thus enabling to reduce acquisitions while preserving image quality and structure.

## Methods

### Linear inverse problems

The acquisition process generates a set of projection images of the object of interest. Each of these images corresponds to a specific angle and can be represented as a set of integrals along straight (parallel) lines, determined by the geometry of the system. The relation that goes from the underlying distribution (object) to the angular projections is called the *Radon* transform, which is a linear operator. The problem of reconstructing images from its angular projections (sinogram) consists in the determination of the pseudo-inverse Radon transform. For the case of reconstructing an image or slice *x* over a plane from the 2D sinogram whose value is equal to the line integral of *x* over that line^11^. Radon’s theory provides an analytical framework for solving this inversion problem, commonly known as the filtered *backprojection* (FBP).

Let ${\textbf{x}}$ be an image over which a forward operator ${\mathscr {A}}$ acts upon. The idea behind compressed sensing is to recover a discrete approximation ${\textbf{x}}\in {\mathbb {R}}^{\text{N}}$ of the original image from a vector of undersampled measurements ${\textbf{b}}$, where ${\mathscr {A}}({\textbf{x}}) = {\textbf{b}}\in {\mathbb {R}}^{\text{M}}$.

In linear inverse problems, the forward operator ${\mathscr {A}}$ can be written as a matrix ${\textbf{A}}\in {\mathbb {R}}^\mathrm {M\times N}$ and, in the case of ${\textbf{A}}$ being a rectangular matrix, the recovery of ${\textbf{x}}$ from ${\textbf{b}}$ is ill-posed. In the case of the tomographic acquisition, we consider the sampling matrix ${\textbf{S}}$, which selects rows of the sinogram, obtaining ${\textbf{A}} = {\textbf{S}}{\textbf{R}}$, where ${\textbf{R}}$ is the matrix representation of the Radon forward operator.

The reconstruction problem can be written in a regularised least-squares form:

1 $$\begin{aligned} \mathbf {x_{rec}} = \arg \min _{{\textbf{x}}} ||{\textbf{A}}{\textbf{x}} - {\textbf{b}}||^2_2 + \lambda {\mathscr {R}}({\textbf{x}}), \end{aligned}$$

where $\lambda$ is the regularisation parameter and ${\mathscr {R}}:{\mathbb {R}}^{\text{N}} \rightarrow {\mathbb {R}}_{\ge 0}$ is a sparsity-promoting regularisation function. While typical choices for regularisation are based on total variation^12^ and wavelets^13^, the usage of a CNN estimator of aliasing in the image reconstruction has been proposed as a novel way to regularise linear inverse problems. In comparison to a computationally expensive usage of neural networks as a learnable mapping between the acquisition ${\textbf{b}}$ and the desired image ${\textbf{x}}$^14^, the physics model in combination with a CNN as a regulariser reduces dramatically the network receptive field size needed whilst providing good quality results. We reformulate (1) as an unrolled network with a CNN regularisation scheme as:

2 $$\begin{aligned} \mathbf {x_{rec}} = \arg \min _{{\textbf{x}}} ||{\textbf{A}}{\textbf{x}} - {\textbf{b}}||^2_2 + \lambda ||{\textbf{x}}-{\mathscr {D}}_{\mathbf {\theta }}({\textbf{x}})||^2_2, \end{aligned}$$

where ${\mathscr {D}}_{\mathbf {\theta }}({\textbf{x}})$ is the denoised version of ${\textbf{x}}$, i.e., after artefact and noise removal, with $\theta$ being the trainable parameters of the neural network. The first term is the data consistency term, which enforces consistency between the measured data and model prediction. Using the unrolled formulation presented in^9^, Eq. (2) can be approximated by K iterations of a two-step alternating algorithm:

3 $$\begin{aligned} {\textbf{x}}_{k}&= ({\textbf{A}}^{\textbf{T}}{\textbf{A}}+\lambda {\textbf{I}})^{-1}({\textbf{A}}^{\textbf{T}}{\textbf{b}}+\lambda {\textbf{z}}_{k-1}), \end{aligned}$$

4 $$\begin{aligned} {\textbf{z}}_k&= {\mathscr {D}}_{\mathbf {\theta }}({\textbf{x}}_k). \end{aligned}$$

In order to determine the weights $\mathbf {\theta }$ of the CNN, the simplest training strategy consists in minimising a mean square error (MSE) cost function ${\mathscr {C}}_{{\text{MSE}}}$, which can be calculated over the pairs of target ${\textbf{t}}^{(i)}$ and output images ${\textbf{x}}^{(i)}_K$, with $i = {1,..., P}$, being *P* the number of examples presented to the network in the training phase:

5 $$\begin{aligned} {\mathscr {C}}_{{\text{MSE}}} = \sum _{i=0}^{P} ||{\textbf{t}}^{(i)}-{\textbf{x}}^{(i)}_{\text{K}}||^2. \end{aligned}$$

### Compared reconstruction methods

The performance of the proposed method was compared qualitatively and quantitatively against three different reconstruction methods, as described below and in Table 1.

**Table 1:** Reconstruction methods under study.

	Reconstruction type	Regularisation	Hyperparameters	Reconstruction time (per slice)	Trainable parameters (detector size $\sim$**100 pixels)**
**FBP**	Analytic	–	–	$\sim$100 ms (CPU) $\sim$1 ms (GPU)	–
**TwIST**	Iterative	Total Variation (TV)	$\lambda = 0.01$^2^	$\sim$10 s (CPU) $\sim$30 ms (GPU)	–
**U-Net**	Supervised learning	–	–	$\sim$10 ms (GPU)	$\sim$10 M
**ToMoDL**	Model-based learning	Convolutional neural network	${\text{K}} = 8$ $\mathrm {N_L} = 8$ Learnable $\lambda$^9^	$\sim$100 ms (GPU)	$\sim$200.000

**FBP** is a widely used method for tomographic reconstruction. The method involves filtering the data in the frequency domain and then backprojecting the filtered data onto the 3D volume. The filter used in FBP is typically a ramp filter, which amplifies high-frequency components of the data. FBP is computationally efficient and works well for simple geometries, such as parallel-beam tomography.

**TwIST (Two-step Iterative Shrinkage and Thresholding)** is an iterative method for tomographic reconstruction, which involves iteratively solving a convex optimszation problem such as (1) using the shrinkage and thresholding technique^15^ for a 2D slice. In this work, we chose to minimise the total variation norm as our regularising function. TwIST can handle a wide range of geometries and produces high-quality reconstructions. However, it is computationally expensive and requires careful tuning of parameters^2^.

**U-Net** is a deep learning architecture for tomographic reconstruction that uses a U-shaped network with skip connections^16^. The proposed network by Davis et al.^7^ processes undersampled FBP reconstructions and outputs streak-free 2D images. The skip connections help preserve fine details in the reconstruction and the network can handle complex geometries and noisy data. While reconstruction times for this approach are short, making it suitable for real-time imaging, training a U-Net requires a large amount of data.

**Figure 1:**
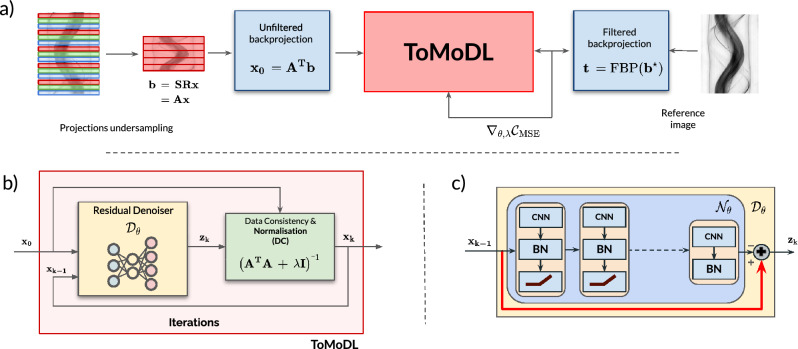
ToMoDL algorithm for optical projection tomography reconstruction. (**a**) To reproduce an accelerated environment, we apply the unfiltered backprojection to a subset of angular-equispaced projections. ToMoDL is trained by comparing the undersampled reconstructions against the analytical solution (FBP) obtained from the fully sampled sinogram, $\mathbf {b^\star }$. (**b**) End-to-end ToMoDL is formed by a residual CNN acting as regulariser, and a conjugate gradient numerical solution of the linear operator. (**c**) Residual network: $\mathrm {N_L}$ stacked layers for noise estimation and addition operation for removal. The weights block is shared across ${\text{K}}$ iterations.

Our proposed method **ToMoDL**, shown in Fig. 1, is a deep learning-based method for tomographic reconstruction that uses a CNN with a residual learning architecture. This residual architecture helps mitigate the vanishing gradient problem and allows for faster training. ToMoDL can produces high-quality reconstructions with low computational cost and a reduced inference times. Let us note that ToMoDL has a longer reconstruction time than the U-Net method even if the neural network involved is much smaller. This is due to the fact that ToMoDL includes also the data consistency stage of the algorithm that is implemented via the conjugate gradients sub-blocks.

### Implementation details

As described in the previous section, each iteration in the unrolled model-based approach can be separated in two stages: a data consistency step $\textbf{DC}$, ensuring consistency between the acquired data and the measurements generated with the forward model, and a trainable denoising block ${\mathscr {D}}_{\mathbf {\theta }}$. Source code for the presented work can be found on GitHub at https://github.com/marcoso96/ToMoDL and Zenodo at https://doi.org/10.5281/zenodo.10056893.

While MoDL theory for regularised reconstruction has been recently developed and implemented within the field of MRI imaging^9,17^, its potential for tomographic reconstruction is essentially constrained by the computational burden that the Radon transform poses when applied iteratively. To overcome this limitation, our proposed method, ToMoDL, incorporates TorchRadon^18^, a fast differentiable routine for computed tomography reconstruction developed as a PyTorch extension. As backpropagation through the DC block consists in a costly linear operator inversion, an efficient conjugate gradient-based numerical implementation was used to replace the analytical calculation.

The CNN architecture for the denoising block ${\mathscr {D}}_{\mathbf {\theta }}$, displayed in Fig. 1c consists of a residual network^19^ shared across ${\text{K}}$ iterations, where $\mathrm {N_L}$ layers with $3\times 3$ convolutional kernels are stacked (noise learner ${\mathscr {N}}_{\mathbf {\theta }} = {\textbf{x}}-{\mathscr {D}}_{\mathbf {\theta }}({\textbf{x}})$). The first layer is skip-connected to the final one, in order to remove the learned noise from the original image. Each 64-filter layer comprises a convolutional operation followed by a non-linear activation function ReLU (rectified linear unit)^20^ in all but the last layer, in order to avoid truncating the negative noise patterns learned.

Batch normalisation was also included for faster and more stable training of the CNN^21^. Due to our memory constraints, we used a limited number of samples per batch (5–10), which did not seem to affect the performance of the network.

We used ${\text{K}}=8$ iterations after we verified that this number is large enough to achieve convergence.

### Training methodology

**Figure 2:**
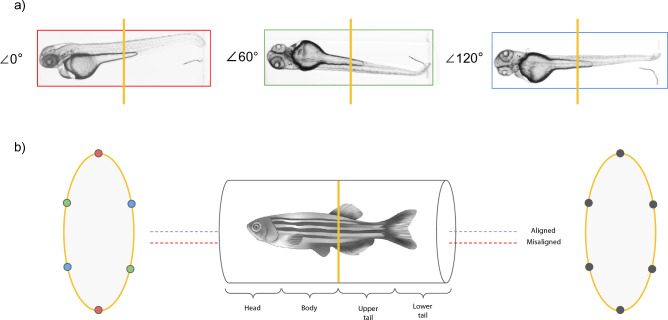
Optical projection tomography setup. (**a**) Projection samples obtained from different angular positions of the detector, where each box colour relates to an angular position of the detector. (**b**) Accelerating OPT consists primarily in using only a fraction (left) of the whole sinogram needed for an streak-free FBP reconstruction. The possible tilt during the acquisition process can be corrected by maximising the variance of the reconstructed image for different positions of the center of rotation.

Here, the training procedure for ToMoDL is described. Input images are obtained by taking equispaced angular projections, as shown in Fig. 2a. The initial angle is chosen randomly for each slice. The undersampled image used as input for the network is obtained using the unfiltered backprojection method (Fig. 2b), while fully sampled sinograms (denoted as $\mathbf {b^\star }$) are reconstructed via the FBP algorithm and fed as reference images for the training phase. As in MoDL, the penalty parameter is considered as a trainable one.

We trained U-Net and ToMoDL networks using a 4-fold cross validation (CV) procedure, where four networks are trained using 9 volumes on each fold (consisting of 880 slices each), leaving the remaining 3 volumes aside for testing. CV allows validating the effect of overfitting on large non-parametric models. Over the 9 volumes, a 80/20 split has been applied for the training and validation sets, respectively. For the FBP and TwIST reconstructions, we proceeded testing over the set of volumes available.

### Zebrafish OPT imaging

Projection data of wild-type zebrafish (*Danio rerio*) at different stages of development ranging from 1 to 5 days post fertilisation were obtained as described in^3^ using 4x objective projections. Using a rotatory cylinder, transmitted projections images were acquired with an angle step of 0.5 degrees. The acquired projections had 700$\times$700 pixels with a resolution of 1.3 μm per pixel. These projections were resampled and normalised so that the FBP projections would have a resolution of $100\times 100$ pixels in order to reduce the computational complexity of the training phase.

For the reconstruction task, we chose 12 volumes from different specimen sections. Tomographic artefacts due to rotation axis misalignment, such as ’double-wall’^22^, were corrected by maximising the variance of the reconstructed images from sinograms modified via rigid registration to different locations of the centre of rotation (Fig. 2b). Zero-padding masks were used in order to undersample the sinogram while retaining its size for a consistent usage of its size throughout the network iterative methodology.

FBP was used to reconstruct images from (pre-processed) fully sampled datasets, to obtain ideal “reference” images. Then, undersampled datasets corresponding to acceleration factors of R = {4, 8, 12, 16, 20, 24, 28} were generated from the fully sampled test datasets and reconstructed using FBP, TwIST, U-NET and ToMoDL. The term acceleration factor indicates that R-times less data was used. It is defined as the ratio of the amount of data required for a fully sampled acquisition to the amount of data collected in an accelerated acquisition. The structural similarity index (SSIM) and peak signal-to-noise ratio (PSNR) were used to quantitatively assess the quality of the image reconstructed using FBP, TwIST, U-NET and ToMoDL in comparison with the reference images.

## Results

### Impact of acceleration factor

Considering the fully sampled FBP reconstruction as the reference image, the analytical (FBP) and iterative (TwIST) solutions present accurate results in terms of peak signal-to-noise ratio (PSNR) and structural similarity index metric (SSIM) for acceleration factors below 10x. In particular for TwIST with TV regularisation, its edge preservation property relates to the high PSNR of its reconstruction. For acceleration factors higher than 10x, streak artefacts induced by undersampled reconstruction arise on FBP reconstructions and total variation regularisation loosely constrains this artefacts.

**Figure 3:**
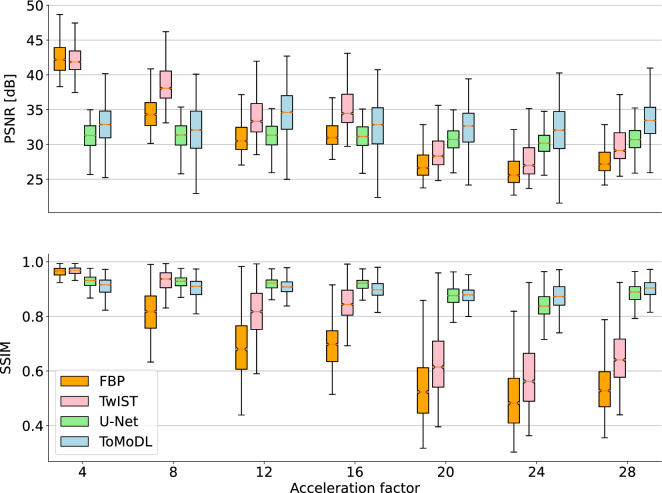
PSNR and SSIM reconstruction quality metrics. The U-Net and ToMoDL approaches both outperform FBP and TwIST for acceleration factors higher than 10x and exhibit similar reconstruction quality metrics. The difference in the PSNR spread is attributed to datasets with small or none structure present.

We can observe on Fig. 3 that, while the PSNR for the U-Net and the proposed ToMoDL display similar mean performance, with ToMoDL outperforming all methods for acceleration factors of 20x or higher, the spread of the quantitative metric might indicate that we are dealing with a less robust algorithm. However, it was observed that poor performing reconstructions are found in volumes whose sections barely show any structure, such as the ones from the lower tail (see Supplementary Material, Fig. S1).

Under these settings, both trainable methodologies present a consistently high structural similarity with the reference image. In order to assess the impact of learning from a scarce number of training samples, we performed an exploratory study to evaluate the performance of the trainable methods with a variable number of samples. In the next section we derive suggestive evidence that the U-Net could be overfitting the training dataset, despite our efforts to cross-validate with different volumes.

### Number of training samples impact

**Figure 4:**
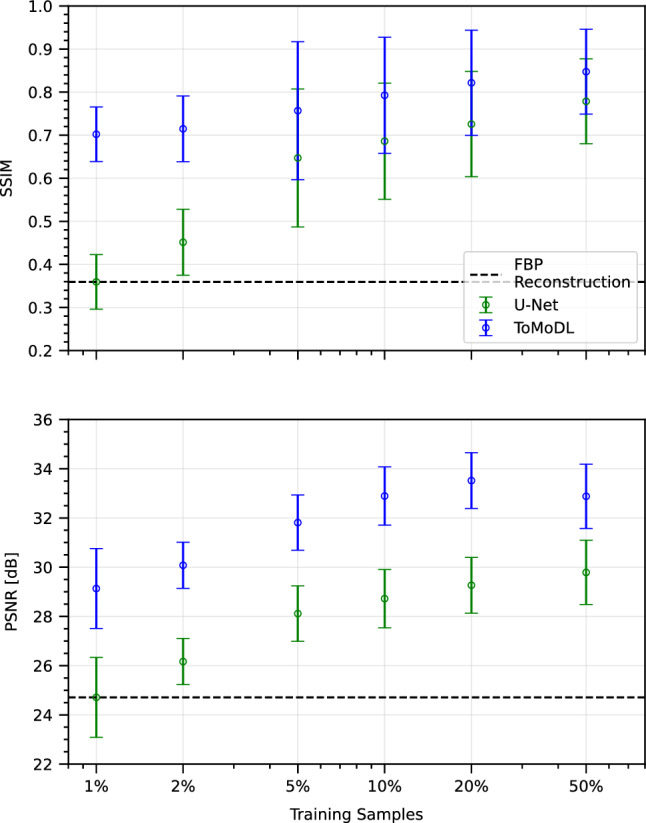
Impact of training samples. In terms of structural similarity (SSIM) and peak signal-to-noise ratio (PSNR), ToMoDL shows a strong generalisation capability for high acceleration factors (20x on display), even for a 1% of the total available training data ($\sim$75 samples).

Figure 4 shows the SSIM and PSNR metrics for the trainable models evaluated on the test volumes of each fold as a function of the number of samples used in the training process, for the specific case of 20x acceleration. It can be seen that ToMoDL achieves high SSIM and PSNR average values of about 0.8 and 33 dB, respectively, even when only 10% of the samples are used for training. The results show a clear preservation of the SSIM while training on a small number of samples, reaching values . Recalling the number of trainable parameters in U-Net ($\approx 10^7$) in comparison to ToMoDL ($\approx 2\times 10^5$), its performance still improves as the number of training samples increases, indicating a poor generalisation capacity which could eventually lead to overfit the presented examples. In contrast, ToMoDL reconstruction metrics peak when a 20% of the total available training data is presented.

Therefore, we observe that iteratively boosting the denoiser network with the underlying reconstruction model not only allows for lower-complexity on its construction, but also for reliable reconstructions with far fewer training examples than in classical denoising strategies. While downsides could be pointed out at the requirement of a fast Radon operator in terms of memory usage, we argue that large non-parametric approaches such as U-Net imposes barely any model constraint with the same computational burden.

### Qualitative comparison

**Figure 5:**
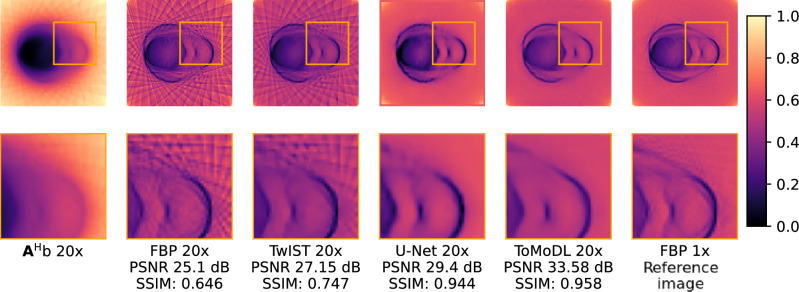
Comparison between OPT reconstruction methods. Images were obtained from a 20x undersampled dataset, ${\mathbf{A}}^{{\text{T}}} {\mathbf{b}}$ and FBP correspond to the standard unfiltered and filtered backprojection methods, respectively. PSNR and SSIM values are shown for the different image reconstruction methods. FBP 1x was used as the reference image (without projection subsampling). An zoomed-in section of the reconstructed structure is displayed in the second row.

Fig. 5 displays different metodologies for reconstructing OPT images with a 20x acceleration factor. The zoomed area (indicated by the box) shows that the U-Net and ToMoDL yield the cleanest results in terms of streaking artefacts, exhibiting a visual quality that is comparable to the fully-sampled FBP reconstruction. The TwIST reconstruction presents a smoothed version of the 20x FBP image, with a higher edge preservation than the U-Net method (see Supplementary Material, Fig. S2, for reconstruction obtained with different acceleration factors).

**Figure 6:**
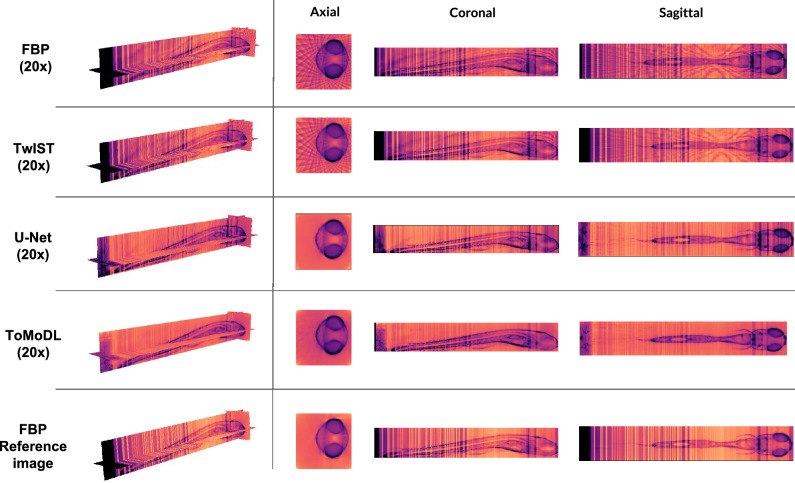
3D reconstruction. Axial, sagittal and coronal slices of a 5-day post fertilisation zebrafish obtained from a 20x undersampled dataset using FBP, TwIST, U-Net and ToMoDL, as well as the reference image reconstructed from the fully sampled sinogram.

For the reconstructions obtained with 20x undersampled data, we can see in Fig. 6 that the 3D volume obtained with ToMoDL has an image quality comparable to that obtained with FBP from fully-sampled data. The latter reconstruction method uses 720 projections whereas a 20x acceleration factor implies that only 36 projections were used in the reconstruction. Artefacts in the reconstruction can be found for high spatial frequency details such as the dorsal region and the organs in the central region. On the other hand, let us note that the ToMoDL reconstruction does not display the vertical streaks that appear in the reference image. These streaks correspond to fluctuations of the mean signal for different slices of the image. As discussed in more detail in the Supplementary Material (Fig. S3), this effect is generated by the registration algorithm. As FBP processes each slice independently, these fluctuations are preserved in the reconstruction. In contrast, our method is able to learn approximately the correct probability distribution of intensities and effectively perform an interpolation that smooths the streaks. This results in a reconstruction that looks visually better than the the reference image. The software Slicer3D was used to generate the 3D cuts for the different reconstructed volumes.

## Discussion

In this work, we proposed ToMoDL, a model-based deep learning reconstruction method, to greatly accelerate OPT acquisitions and reconstructions. Using the MoDL framework as our st arting point, the introduction of a fast Radon operator enabled the reconstruction of high-quality images with two orders of magnitude fewer learnable parameters than in DL methods for OPT. Furthermore, we observed that our ToMoDL implementation can rely on a small dataset ($\sim 75$ samples) to reconstruct images with a competitive performance. To our knowledge, this is the first time that an unrolled network has been applied to OPT, more specifically for reconstructing 3D images from undersampled OPT data in real-time. In the future, we plan to extend ToMoDL to other tomographic imaging modalities, such as Positron Emission Tomography (PET) and X-ray Computed Tomography (CT).

In contrast with MRI reconstruction, whose solution is based on the Fourier transform, the geometrical OPT reconstruction model uses the Radon transform that is not a unitary operator. Because of the lack of conservation of the norm, we found several shortcomings while training the unrolled neural network with conjugate gradient sub-blocks, such as exploding or vanishing gradients. Our proposed solution consists in an internal normalisation after each numerical inversion of the analytical problem. Future work should aim at extending this framework to non-unitary operators, developing theory in order to avoid common problems arising in the intersection of inverse problems and deep learning.

## Conclusion

ToMoDL, a new model-based framework for tomographic reconstruction was presented, developing its application for optical projection tomography. The presented framework shows competitive results in terms of PSNR and SSIM, when compared to the U-Net and TwIST methods, two state-of-the-art methods for OPT reconstruction from undersampled data.

Built on fast differentiable Radon operators, this inverse problem approach enables real-time reconstruction and future work will be researching its extension with different subject-related priors^17^. Moreover, the capability of learning with a small training dataset (less than 100 samples) presents a extensible paradigm for transferring its usage from one specimen to another. Lastly, the proposed method could also be extended to other tomographic imaging modalities, such as x-ray.

### Supplementary Information

Supplementary Information.

## Data Availability

Sample cover data and code are available at https://github.com/marcoso96/ToMoDL and Zenodo at https://doi.org/10.5281/zenodo.10056893.
