# Supplementary material for: Model-based deep learning framework for accelerated optical projection tomography
Source: Sci Rep. 2023 Dec 8;13:21735. doi: 10.1038/s41598-023-47650-3 (PMC10709405; doi:10.1038/s41598-023-47650-3)
Supplement: Supplementary file 1 — Supplementary Information. [file 41598_2023_47650_MOESM1_ESM.pdf]

Supplementary material

Performance according to specimen section

We observed that the performance of the reconstruction method strongly depends on the imaged section of the specimen. For the purpose of this analysis, we divided the volume into four sections: head, body, upper tail and lower tail, as shown in Fig. S1a). In Fig. S1b, for each slice i of an specific volume (3-day post fertilisation *Danio rerio*), we plotted the resulting PSNR of the reconstruction using the undersampled FBP and ToMoDL reconstruction against the corresponding difference of the standard deviation of the intensity between slices i and $i+1$, $\Delta\sigma_I^i$. At a first glance, we observe the poor performance of ToMoDL where less structure can be found, such as the lower tail, whereas the FBP provides a good quality image, as expected. Furthermore, while the intensity variations along slices decrease from the bottom towards the top sections of the specimen, S1b) they do not explain the performance of the method for individual slices.

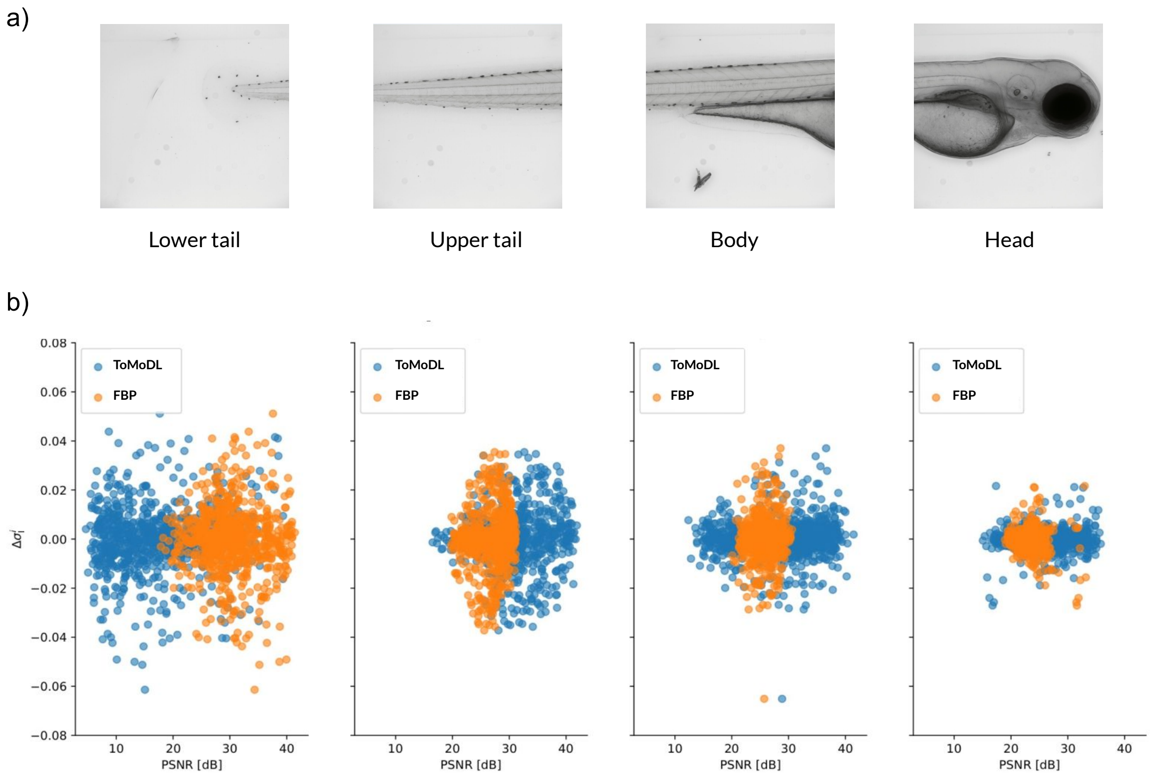

Figure S1. Example projections and volume reconstruction per section. a) For each specimen's section, (head, body, upper and lower tail),**b)** we evaluated the undersampled reconstruction obtained with the FBP and ToMoDL methods for each slice in the corresponding section. Then, we plotted the PSNR against the difference of the standard deviation of the intensity between slices i and $i+1$, $\Delta\sigma_I^i$. We observe that sections with less structure have significantly more intensity variance and higher reconstruction error, although these values are poorly correlated on a slice-by-slice basis.

Qualitative results per acceleration factor

For different acceleration factors, we present the qualitative results for the four compared reconstruction algorithms: FBP, TwIST, U-Net, and our proposed method ToMoDL. We observe that ToMoDL removes streaking artefacts and reconstructs images with high visual quality for all acceleration factors, whereas methods such as TwIST and U-Net display a fairly good performance for acceleration factors up to 16x.

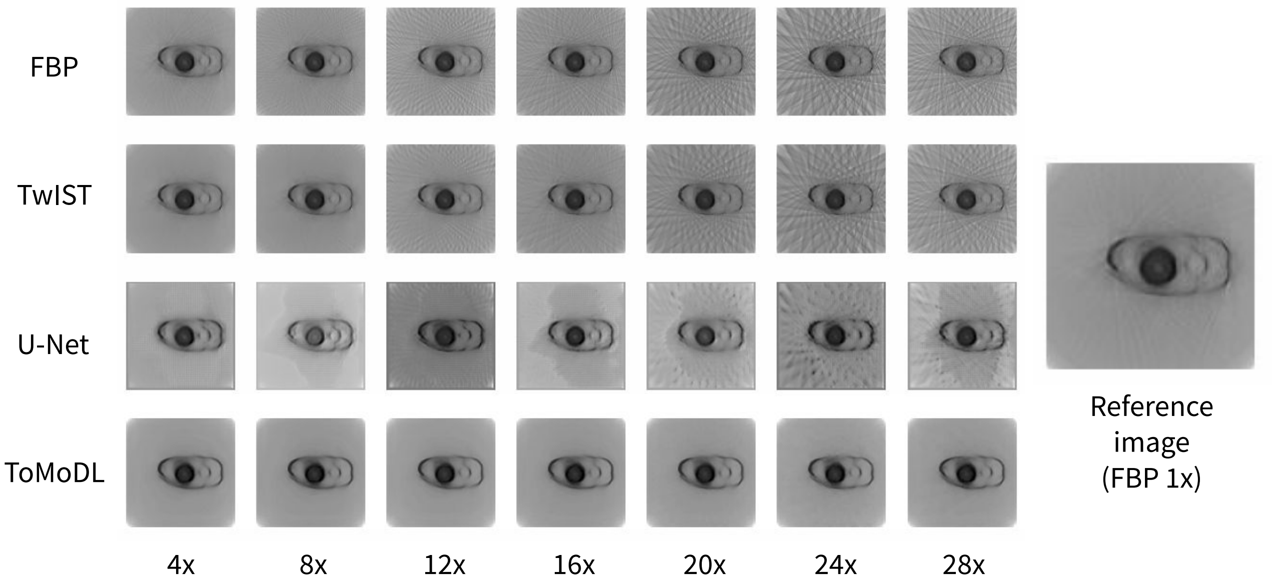

Figure S2. Reconstructions for different acceleration factors. Reconstructions for different acceleration factors. Images (slice of a body section) obtained with FBP, TwIST, U-Net and the proposed ToMoDL from 4x, 8x, 12x, 16x, 20x, 24x and 28x undersampled data. ToMoDL effectively removes streaking artefacts and reconstructs images with high visual quality for all acceleration factors, whereas state-of-the-art methods such as TwIST and U-Net show a good performance for acceleration factors up to 16x. For higher acceleration factors, the streaking artefacts on TwIST and FBP degrade the images and the U-Net images appear blurred.

Artefacts due to the alignment algorithm

The reconstruction of individual 2D slices requires an additional preprocessing step to align the rotation axis of the OPT sample container with respect to the center of the detector, as shown in Fig. S3a. Following the method described in[1] for artefact correction, the most suitable registration shift in the projections (sinograms) space was identified by selecting the reconstructed image with the largest intensity deviation σ_I (blue line in Fig. S3b). As shown in Fig. S3b, a small uncertainty on the shift with respect to the peak produces a strong decrease in the image mean intensity μ_I (yellow line), which in turn results in different gains in the mean intensity of the reconstructed images. We can also observe that both peaks do not necessarily coincide for the optimal shift. Fig. S3b displays three reconstructed planes obtained after applying registration shifts of 10, 12 (optimal shift) and 14 pixels (vertical dotted lines) to show the dependence of the mean intensity of the image on the alignment. The noticeable change in the intensities gives rise to the streaks in the 3D reconstruction (seen as intensity heterogeneities along the z-axis in Fig. 6).

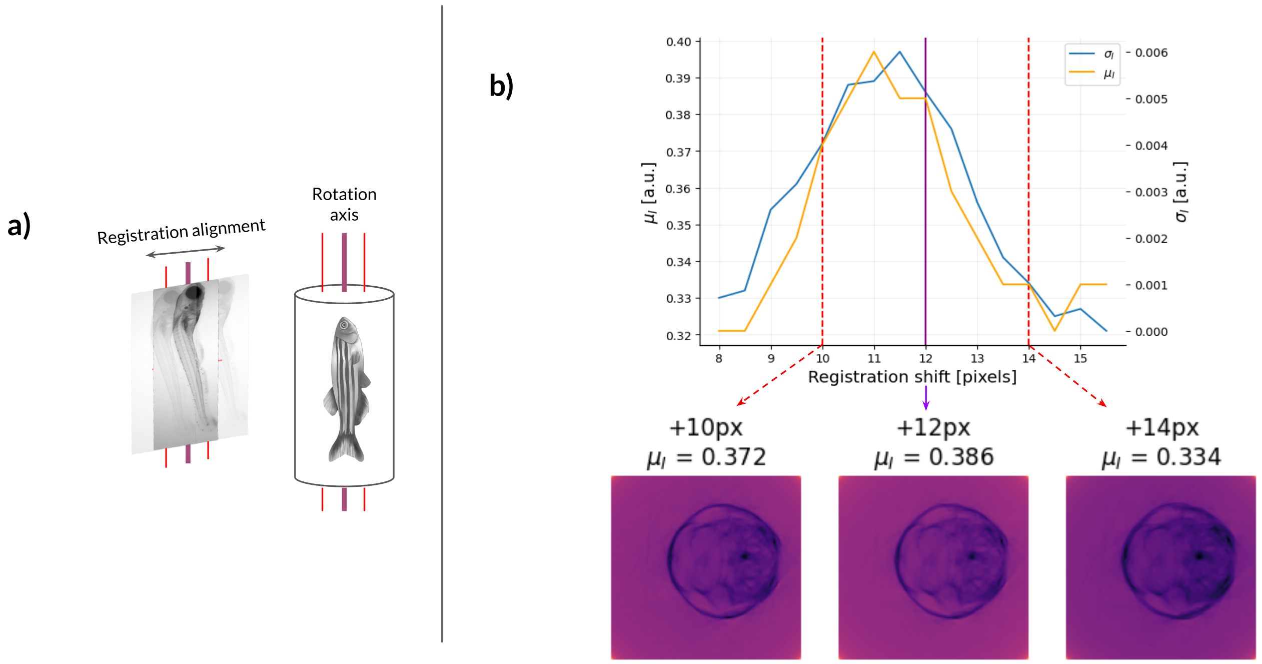

Figure S3. Alignment algorithm. a) The registration algorithm applies shifts in the sinogram space in order to maximize the standard deviation of intensity σ_I (blue line) in the image space. **b)** A small misalignment correlates with a blurring effect on the final reconstructions and with a decrease of the mean intensity of the image μ_I (yellow line). Since in most cases these peaks do not often coincide, abrupt changes of illumination appear along the z-axis of the reconstructed volume.

References

1. Walls, J. R., Sled, J. G., Sharpe, J. & Henkelman, R. M. Correction of artefacts in optical projection tomography. *Phys. Medicine & Biol.* **50**, 4645 (2005).
